# Health impact of intimate partner violence and implication on services in Malaysia

**DOI:** 10.1186/1471-2458-12-S2-A8

**Published:** 2012-11-27

**Authors:** Siti Hawa Ali, Tengku Nur Fadzilah Tengku Hassan, Halim Salleh, Harmy Mohamed Yusoff

**Affiliations:** 1 School of Health Sciences, Health Campus, Universiti of Sciences Malaysia, 16150 Kubang Kerian, Malaysia; 2 Women’s Health Development Unit, School of Medical Sciences, Health Campus, Universiti of Sciences Malaysia, 16150 Kubang Kerian, Malaysia; 3 Department of Community Medicine, School of Medical Sciences, Health Campus, Universiti of Sciences Malaysia, 16150 Kubang Kerian, Malaysia; 4 Department of Family Medicine, School of Medical Sciences, Health Campus, Universiti of Sciences Malaysia, 16150 Kubang Kerian, Malaysia

## Background

Intimate partner violence (IPV) is an emerging public health problem that requires attention within the health sector system. The implications for women include physical injuries and mental health challenges. The purpose of this study was to examine the impact on health of women experiencing violence and to create awareness among health care providers.

## Materials and methods

A cross-sectional study was conducted from May 2009 until May 2010 among women experiencing IPV who sought help from thirteen women non-governmental organizations in Malaysia. A total of 316 women were randomly selected and interviewed using a translated and validated WHO Multi-country Study on Women’s Health and Life Experiences questionnaire. Data was analyzed using PASW version 18. The disseminations of the results from this study were done through meetings and dialogues with health service care providers.

## Results

Most of the women experienced emotional violence (96.8%) followed by physical (91.5%) and sexual violence (58.9%). About 56% of the women experienced all three types of violence. A majority (82.4%) reported that they had some kind of injuries and these were significantly associated with IPV (p<0.05). Furthermore, women reported high level of emotional distress resulting from IPV. Due to the severity of injuries, women ended up seeking health care services (51%) and reported 79.6% of satisfaction with the services provided. A majority of them reported that they had been asked by health care worker about the real cause of the injuries.

## Conclusion

Women suffer physical and mental health consequences due to IPV. As a critical institution that assists survivors of IPV, the health sector has taken necessary measures such as development of services for IPV within primary and secondary health care settings. However, it is pertinent for the health care sector to improve their awareness through better understanding of the issues as well as developing innovative measures for screening, treatment and referral systems.

